# Characterization of CD4 T Cell Epitopes of Infliximab and Rituximab Identified from Healthy Donors

**DOI:** 10.3389/fimmu.2017.00500

**Published:** 2017-05-05

**Authors:** Moustafa Hamze, Sylvain Meunier, Anette Karle, Abdelaziz Gdoura, Amélie Goudet, Natacha Szely, Marc Pallardy, Franck Carbonnel, Sebastian Spindeldreher, Xavier Mariette, Corinne Miceli-Richard, Bernard Maillère

**Affiliations:** ^1^CEA-Saclay, Institut de Biologie et Technologies, Université Paris-Saclay, Gif sur Yvette, France; ^2^Novartis Pharma AG, Basel, Switzerland; ^3^INSERM UMR 996, Faculté de Pharmacie, Université Paris-Sud, Chatenay Malabry, France; ^4^Service de gastro-entérologie, Hôpitaux Universitaires Paris-Sud, Le Kremlin-Bicêtre, France; ^5^INSERM UMR 1184, Assistance Publique-Hôpitaux de Paris, Service de Rhumatologie, Hôpitaux Universitaires Paris-Sud, Université Paris-Sud, Le Kremlin-Bicêtre, France

**Keywords:** therapeutic antibodies, immunogenicity, antidrug antibody, CD4 T cell epitopes, healthy donors, infliximab, rituximab, MHC-associated peptide proteomics

## Abstract

The chimeric antibodies anti-CD20 rituximab (Rtx) and anti-TNFα infliximab (Ifx) induce antidrug antibodies (ADAs) in many patients with inflammatory diseases. Because of the key role of CD4 T lymphocytes in the initiation of antibody responses, we localized the CD4 T cell epitopes of Rtx and Ifx. With the perspective to anticipate immunogenicity of therapeutic antibodies, identification of the CD4 T cell epitopes was performed using cells collected in healthy donors. Nine T cell epitopes were identified in the variable chains of both antibodies by deriving CD4 T cell lines raised against either Rtx or Ifx. The T cell epitopes often exhibited a good affinity for human leukocyte antigen (HLA)-DR molecules and were part of the peptides identified by MHC-associated peptide proteomics assay from HLA-DR molecules of dendritic cells (DCs) loaded with the antibodies. Two-third of the T cell epitopes identified from the healthy donors stimulated peripheral blood mononuclear cells from patients having developed ADAs against Rtx or Ifx and promoted the secretion of a diversity of cytokines. These data emphasize the predictive value of evaluating the T cell repertoire of healthy donors and the composition of peptides bound to HLA-DR of DCs to anticipate and prevent immunogenicity of therapeutic antibodies.

## Introduction

Therapeutic antibodies have become a major strategy in oncology and clinical immunology. However, a large subset of patients develops neutralizing antidrug antibodies (ADAs) that reduce their therapeutic efficacy (1) and induce allergic responses (2). As immunological tolerance is expected to regulate the response to self-sequences (3), almost all therapeutic antibodies are humanized, with various degrees of antibody humanization (4). Chimeric antibodies are humanized on their constant parts only, their variable regions remaining of murine origin, while humanized antibodies are also modified in the framework (FR) regions of the variable parts. Fully human antibodies are mostly produced by *in vitro* selection of antibodies encoded by human immunoglobulin genes or by immunization of Ig-humanized mice. Nevertheless, none of these approaches fully guarantee the absence of immune responses. Chimeric antibodies such as rituximab (Rtx) (5–7) and infliximab (Ifx) (8–10) are known to elicit specific ADAs in multiple patients, generally associated with reduced clinical efficacy. Similarly, the humanized antibodies alemtuzumab (11) and vedolizumab (12) and the fully human adalimumab (10, 13, 14) are known to generate ADAs in many patients. While humanization of the constant parts of therapeutic antibodies clearly reduces ADA responses, the benefits of humanization of the variable parts remain controversial (15), reflecting the lack of knowledge about the molecular determinants contributing to immunogenicity of therapeutic antibodies (16).

Immunogenicity of antibodies mainly relies on the presentation of antibody-derived peptides displayed on APCs and their capacity to stimulate specific CD4 T lymphocytes. CD4 T lymphocytes participate to the immune response to therapeutic proteins (16–20). However, T cell epitopes contained in marketed therapeutic proteins are largely unknown, although localization of T cell epitopes could help to mitigate immunogenicity by removing them from the initial sequence (19, 21). As T cell epitopes bind to human leukocyte antigen (HLA) class II molecules, locating HLA class II binding peptides could serve as a first step in the evaluation of the immunogenic potential of therapeutic proteins (22–24). However many good peptide binders to HLA molecules do not necessarily elicit a T cell response, especially those derived from self-proteins as many self-reactive T cells are eliminated by central tolerance. Therefore, T cell assays have been established using cells collected from healthy donors (25–29). As healthy donors have never been exposed to therapeutic proteins, T cell assays aim to detect low-frequency specific naïve T cells, assuming that the assays reproduce the memory T cell response that occurs after injection of the therapeutic proteins in the body. However, the relevance of T cell epitopes identified from healthy donors accounting for the T cell response in patients has never been formally demonstrated for therapeutic antibodies.

We therefore investigated the T cell response to the immunogenic chimeric antibodies Rtx and Ifx. Rtx is specific for CD20, a surface marker of B cell lymphocytes and is a B cell-depleting therapeutic antibody approved for the treatment of many lymphomas, leukemias, and autoimmune disorders (5–7, 30). Ifx targets TNF-α and has been proven highly effective in the treatment of inflammatory diseases (8–10). Both antibodies are immunogenic in many patients suffering from inflammatory diseases with an immunogenicity incidence ranging from 10 to 60% (5–10). In this study, we identified the T cell epitopes in the variable parts of Rtx and Ifx using cells collected from healthy donors and evaluated their capacity to stimulate T cells collected from patients with ADA. We characterized naturally presented HLA class II peptides from human dendritic cells (DCs) loaded with the antibodies and evaluated the binding affinities of overlapping peptides to HLA class II molecules. We therefore established a detailed map of the T cell epitopes of Rtx and Ifx, which helped to understand the origin of their immunogenicity.

## Materials and Methods

### Proteins and Peptides

Keyhole limpet hemocyanin (KLH) was purchased from Thermo Fisher Scientific (Brebières, France). Rtx (Mabthera^®^) was purchased from Roche (Neuilly, France) and Ifx (Remicade^®^) from Centocor (Horsham, PA, USA). Peptides were purchased from Pepscan (Lelystad, The Netherlands).

### Characterization of Antibody-Specific CD4 T Cell Lines

Peripheral blood mononuclear cells (PBMCs) were obtained from blood cells collected at the Etablissement Français du Sang (EFS, Rungis, France), as buffy coat preparations from anonymous healthy donors who gave informed consent, in accordance with EFS guidelines. Antibody-specific CD4 T cell lines were generated as described previously (28). DCs were produced from plastic-adherent cells of PBMCs, while CD4 T cells were isolated from PBMCs by using magnetic microbeads (Miltenyi Biotech, Paris, France). DCs were loaded overnight at 37°C with the therapeutic antibody or with KLH used as a positive control (1 µM) and matured with lipopolysaccharide (1 µg/mL). CD4 T cells (200,000/w) were stimulated by protein-loaded DCs (20,000/w) and cultured during 21 days (28). Their peptide specificity was tested by interferon-γ (IFN-γ) ELISPOT (28). Spot number was determined by the AID ELISPOT Reader System (AID). CD4 T cell lines were considered as specific when a spot count was twofold higher in the presence of the protein or the peptide than in their absence, with a minimal difference of 25 spots.

### HLA-DR-Specific Binding Assays

Human leukocyte antigen-DR molecules were immunopurified from homozygous EBV B lymphoblastoid cell as previously reported (31, 32). Binding of the Rtx and Ifx peptides to HLA-DR molecules was assessed by competitive ELISA, as previously described (33). A strong binder to each HLA class II molecule was introduced in each assay as reference (33). Data were reported as relative affinity corresponding to the ratio of the IC_50_ of the tested peptide to the IC_50_ of the reference peptide. Means were calculated from at least two independent experiments.

### MHC-Associated Peptide Proteomics (MAPPs) Assay

Human leukocyte antigen-DR-associated peptides from DCs loaded with Ifx or Rtx were identified *via* the MAPPs assay as described previously (34–37). Briefly, CD14 positive mononuclear cells were purified from PBMCs isolated from human buffy coats sampled from consented healthy donors (Blood Donation Center Bern, Bern, Switzerland) and differentiated into immature DCs (37). Immature DCs were matured by adding lipopolysaccharide (1 µg/mL, Sigma) and loaded separately with one of the chimeric antibodies. After incubation for 24 h at 37°C and 5% CO_2_, DCs were harvested, washed in PBS, and lysed in hypotonic buffer containing 1% Triton X-100. After immunoprecipitation of HLA-DR molecules with Mab L243-conjugated beads, peptides were eluted from HLA-DR molecules by adding 0.1% trifluoroacetic acid (Fluka, Buchs, Switzerland) at 37°C and lyophilized using an Eppendorf Concentrator 5301 (Eppendorf AG, Hamburg, Germany). Lyophilized peptides were resuspended in hydrophilic buffer containing 5% acetonitrile and 1.1% formic acid. Peptide composition was analyzed by liquid chromatography (nano capillary system, Dionex Corporation, Sunnyvale, CA, USA) on a self-packed fused-silica C18 reversed-phase nano-high-performance liquid chromatography column connected to a mass spectrometer (Q-Exactive, Thermo, CA, USA) *via* electrospray ionization (LC–ESI–MS/MS). Peptides were identified *via* a database search approach using the SEQUEST algorithm as detailed (37).

### Evaluation of the T Cell Response to Ifx and Rtx Peptides in Immunized Patients

Blood samples from patients immunized against Rtx or Ifx were collected at Bicêtre Hospital (Le Kremlin-Bicêtre, France) in accordance with French law and after approval by the patients. Levels of circulating Ifx or Rtx and of ADAs specific for each antibody were quantified using bridging ELISA kits (Theradiag, Croissy Beaubourg, France). PBMCs were seeded at 5 M/mL with peptides (10 µg/mL) in RPMI 1640 medium supplemented with 10% SAB, 50 IU/mL IL-2 (R&D), 1,000 U/mL IL-4 (R&D), and 1 µg/mL anti-CD28 (Miltenyi). The culture medium was changed every 2–3 days. Cells were harvested at day 10 and incubated with individual peptides (10 µg/mL) in AIM-V supplemented with 0.5 ng/mL IL-7 at 37°C in Multiscreen 96-well plates (Merck Millipore) previously coated overnight at 4°C with 4 µg/mL anti-human IL-5 MAb (TRFK5, Mabtech). After 48 h incubation, supernatants were collected, and IL-5 secretion was detected by successive addition of mouse biotinylated anti-human IL-5 Mab (TRFK4, Mabtech), Extravidin conjugate, and BCIP substrate (Sigma) as described above for IFNγ ELISPOT. Spots were counted with a computer-assisted video image analyzer (AID, Strassberg, Germany). A positive value was assigned to culture wells with a spot count that was twofold higher in the presence of the peptide than in its absence, with a minimal difference of 25 spots and positivity in Student’s *t*-test (*P* < 0.05). Supernatants of IL-5 ELISPOT plates were submitted to Luminex cytokine assays (R&D) for six different cytokines (IL-2, IL-4, IL-10, IL-13, IL-17, and IFN-γ) according to the manufacturer’s instructions (R&D). Sensitivity of detection ranged from 0.4 to 2 pg/mL, except for IL-4 (9.3 pg/mL) and IL-13 (36.6 pg/mL). Cytokine concentrations lower than the lower limits of detection were reported as undetectable. A positive value was assigned to culture wells with a concentration that was twofold higher in the presence of the peptide than in its absence and positivity in Student’s *t*-test (*P* < 0.05).

## Results

### Ifx- and Rtx-Specific T Cell Epitopes Were Identified in the Variable Domains Using Cells Collected in Healthy Donors

**Figure 1:**
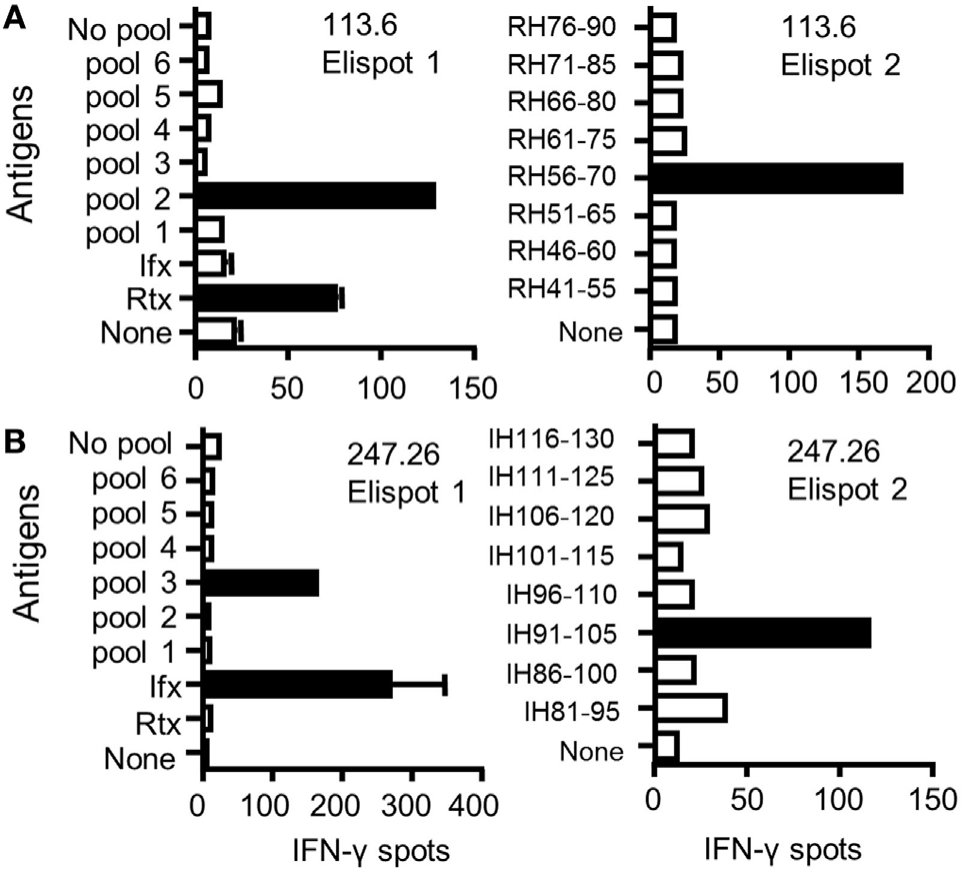
**Identification of CD4 T cell epitopes of infliximab (Ifx) and rituximab (Rtx) from healthy donors**. CD4 T cell lines were generated *in vitro* by four weekly rounds of stimulation with autologous dendritic cells (DCs) loaded with either Rtx or Ifx. Specificity of CD4 T cell lines #113.6 raised against Rtx **(A)** and #247.26 raised against Ifx **(B)** was analyzed by interferon-γ ELISPOT. T cells were incubated with autologous DCs alone (none) or with DCs previously loaded with each antibody (3 µM) or with autologous unloaded peripheral blood mononuclear cells (PBMCs) (no pool) or PBMCs loaded with a pool of peptides (10 µg/mL) (left panels) and with individual peptides (right panels).

**Figure 2:**
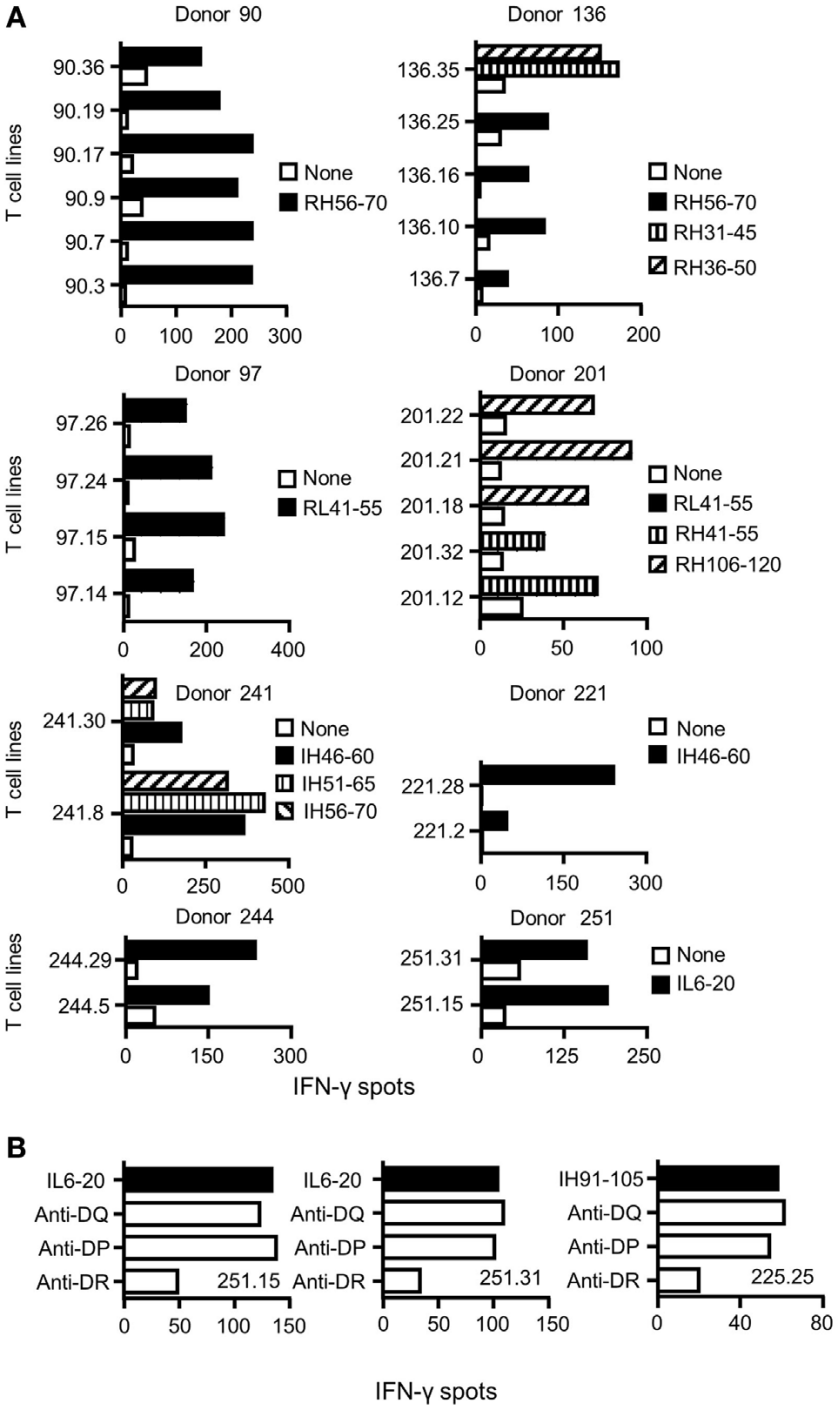
**Specificity and restriction of T cell lines generated from healthy donors**. **(A)** CD4 T cell lines were generated *in vitro* by four weekly rounds of stimulation with autologous dendritic cells loaded with either rituximab (Rtx) or infliximab (Ifx). T cells were incubated with autologous unloaded peripheral blood mononuclear cells (PBMCs) (none) and with PBMCs loaded with individual peptides. Activation of the T cells was evaluated by interferon-γ (IFN-γ) ELISPOT, and peptide specificity was confirmed in two independent experiments. Each panel reports the peptide-specific T cell lines found from one donor. Cells from donors 90, 97, 136, and 201 were used to evaluate the T cell response to Rtx, while cells from donors 221, 241, 244, and 251 were used for Ifx. **(B)** For inhibition assays, anti-HLA-DR (L243), -DQ (SPVL3), and -DP (B7/21) antibodies were added at 10 µg/mL to the IFN-γ ELISPOT.

**Figure 3:**
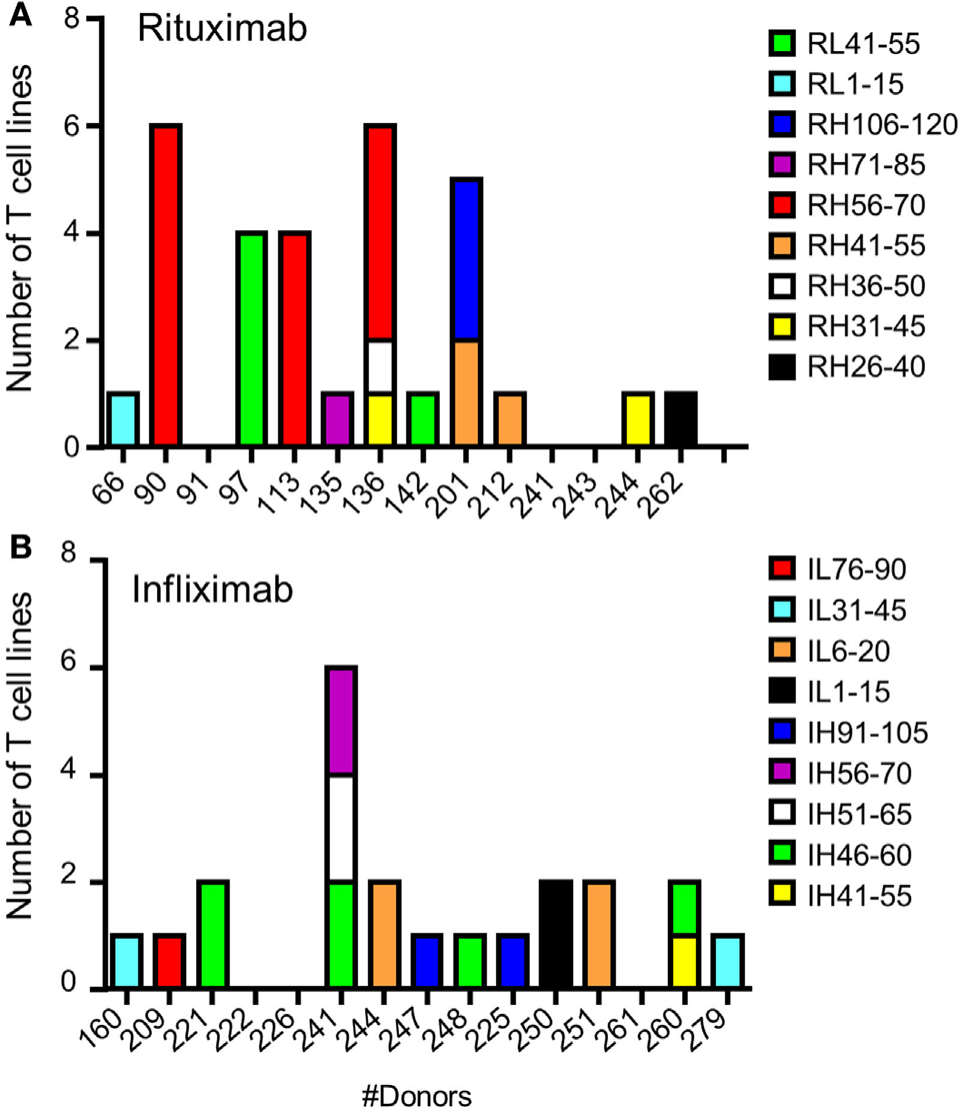
**Specificity for rituximab (Rtx) and infliximab (Ifx) peptides of the CD4 T cells preexisting in healthy donors**. T cell lines were generated, and their peptide specificity was assessed as described in the legend of Figure 1 using cells collected from 14 and 15 donors for Rtx and Ifx, respectively. Donors are identified by a number. The number of T cell lines specific for Rtx **(A)** and Ifx **(B)** is reported for each donor.

To identify CD4 T cell epitopes of Ifx and Rtx, we constituted 2 panels of 15 healthy donors on the basis of the frequency of HLA-DRB1 alleles in Europe (Table S1 in Supplementary Material). From the PBMCs of these donors we derived CD4 T cell lines specific for Rtx and Ifx by 4 weekly rounds of stimulation of the CD4 T cells by DCs loaded with either Rtx or Ifx. CD4 T cell lines were also raised against KLH to assess the ability of the donors to generate specific T cell responses (Figure S2 in Supplementary Material). Each independent T cell line (CD4 T cells present in a single well) was evaluated for its specificity using pools of 45 overlapping peptides encompassing the entire variable regions (VH and VL) of the therapeutic antibodies (Tables S3 and S4 in Supplementary Material), individual peptides, and DCs loaded with the therapeutic antibodies (Figure 1). As an example, T cell line 113.6 from donor #113 was specific for Rtx but not for Ifx and reacted with the pool 2 of Rtx peptides in the first ELISPOT and with the peptide RH56–70 in the second one (Figure 1A). In contrast, T cell line 247.26 from donor #247 was specific for Ifx but not for Rtx and reacted with the pool 3 of Ifx peptides and the peptide IH91–105 (Figure 1B). Each healthy donor gave rise to a variable number of antibody-specific T cell lines as shown for multiple donors (Figure 2A). HLA restriction was evaluated for T cell lines of donors #225 and #251 by inhibition experiments with anti-HLA class II antibodies. These T cell lines appeared to be restricted to HLA-DR molecules and not to HLA-DP or DQ (Figure 2B). Together, 11 out of 14 donors generated a CD4 T cell response to Rtx peptides, and 12 out of 15 donors generated a CD4 T cell response to Ifx peptides (Figure 3). A CD4 T cell response specific for each antibody was sustained by nine different T cell epitopes found in the variable regions of both light and heavy chains. The Rtx peptide RH56–70 was common to three donors, while RH31–45, RH41–55, and RL41–55 were shared by two donors (Figure 3A). Four donors shared the same peptide Ifx IH46–60, while peptides IH91–105, IL31–45, and IL6–20 were common to two donors (Figure 3B).

### Binding of Ifx and Rtx Peptides to Purified HLA Class II Molecules

**Figure 4:**
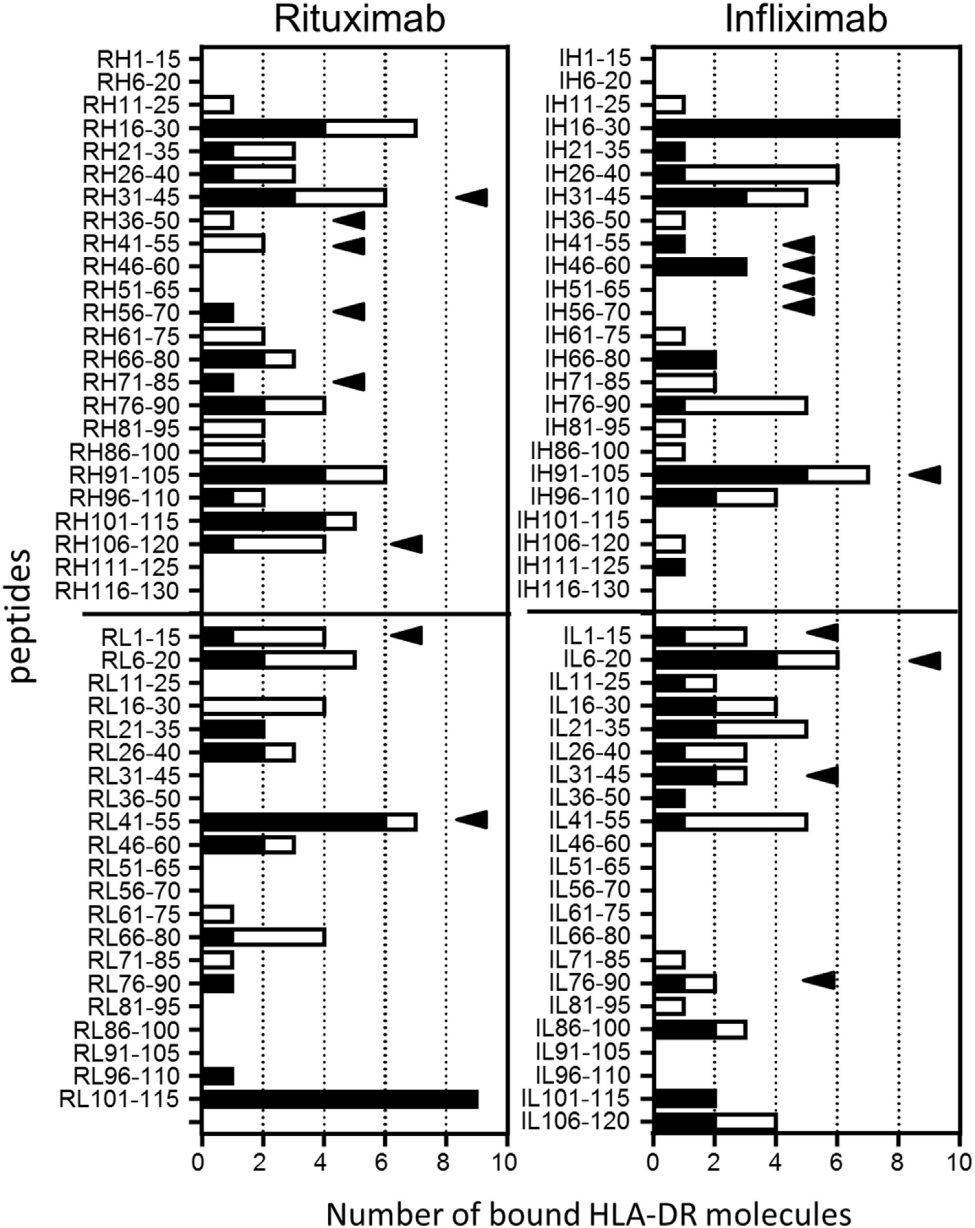
**Binding of rituximab (Rtx) and infliximab (Ifx) overlapping peptides to common human leukocyte antigen (HLA)-DR molecules**. Overlapping 15-mer peptides covering the whole sequence of the variable parts of Rtx and Ifx were submitted to competitive ELISA specific for the following HLA-DR molecules: DRB1*01:01, *04:01, *11:01, *07:01, *01:01, *03:01, *09:01, *1301, *1501, DRB3*01:01, DRB4*01:01, and DRB5*01:01. Data are reported as number of bound HLA-DR molecules for each peptide. Relative affinity below 20 corresponds to strong binding (black), while moderate binding is in the range of 20–100 (blank). Black arrows: CD4 T cell epitopes.

The two sets of overlapping peptides were submitted to 11 different binding assays specific for common HLA-DR alleles (Figure 4). These alleles comprise eight molecules encoded by the HLA-DRB1 (01:01, 03:01, 04:01, 07:01, 09:01, 11:01, 13:01, and 15:01) gene and three second HLA-DR molecules (DRB3*01:01, DRB4*01:01, and DRB5*01:01). Binding activities were found all along the variable sequences of both antibodies. Multiple peptides bound with strong or moderate affinity to multiple HLA-DR molecules and comprised several CD4 T cell epitopes (RH31–45, RH106–120, RL1–15, RL41–55, IH91–105, and IL6–20). As shown Table 1, a majority of CD4 T cell epitopes exhibited a strong or moderate binding to at least one of the HLA-DR molecules of the donors responding to them. It is of note that the peptide RH56–70 was highly specific for the HLA-DR11 molecule and generated a T cell response in three HLA-DR11 donors. In contrast, the peptides RL41–55 and IH46–60 bound to 6 and 3 HLA-DR molecules, respectively, and concomitantly stimulated T cells in 2 and 4 donors, respectively. In summary, most of the T cell epitopes are associated with strong or moderate peptide binding to HLA-DR molecules but not all of the strong binders lead to T cell stimulation.

**Table 1:** **Binding of infliximab and rituximab CD4 T cell epitopes to human leukocyte antigen (HLA)-DR molecules found in responding healthy donors**.

Peptides	HLA molecules						HLA haplotypes of responders			
	DR1	DR4	DR7	DR9	DR11	DR15				
RH26–40	216	**88**	**32**	177	196	**0.4**	DR1/DR8			
RH31–45	**2**	nd	**2**	**60**	**2**	122	DR11/DR14	DR1/DR13		
RH36–50	**32**	156	250	589	378	>1,826	DR11/DR14			
RH41–55	**45**	>462	3,341	106	463	**71**	DR13/DR16	DR15/DR15		
RH56–70	>2,404	211	>28,677	12,500	**13**	>1,826	DR7/DR11	DR11/DR14	DR3/DR11	
RH71–85	**4**	>462	12,500	816	ND	467	DR3/DR15			
RH106–120	**58**	>462	**40**	**100**	>37,796	**11**	DR13/DR16			
RL1–15	20	**90**	**10**	**33**	447	117	DR9/DR13			
RL41–55	**1**	**0.1**	**0.7**	**2**	**5**	**12**	DR4/DR7	DR11/DR15		

IH41–55	2,760	10,000	22,727	>4,979	**11**	238	DR11/DR13			
IH46–60	200	2,424	**5**	657	**5**	**0.4**	DR1/DR7	DR4/DR15	DR7/DR11	DR11/DR13
IH51–65	30,000	25,714	29,545	824	1,479	2,000	DR4/DR15			
IH56–70	3,333	1,863	6,818	962	252	>1,335	DR4/DR15			
IH91–105	**6**	**10**	**9**	**48**	**3**	**13**	DR1/DR13	DR11/DR16		
IL1–15	**11**	>10,526	**35**	474	7,500	**24**	DR3/DR7			
IL6–20	**17**	25,714	**45**	**21**	3,260	**2**	DR7/DR9	DR1/DR13		
IL31–45	183	**5**	3,015	5,556	362	**3**	DR1/DR4	DR3/DR4		
IL76–90	20,000	>10,526	>12,205	>4,979	>11,604	**27**	DR4			

*Results are expressed as a relative binding activity (ratio of the IC_50_ of each peptide by that of the reference peptide of the tested HLA molecule). Numbers in red (relative affinities less than 20): high binders. Numbers in green (relative affinities in the range of 20–100): moderate binders. Concordance between HLA allotypes of the donors responding to a T cell epitope and good or moderate binding of the T cell epitope is indicated by frames*.

### CD4 T Cell Epitopes Are Retrieved from Elution Experiments of Naturally Presented Peptides of Rtx and Ifx

**Figure 5:**
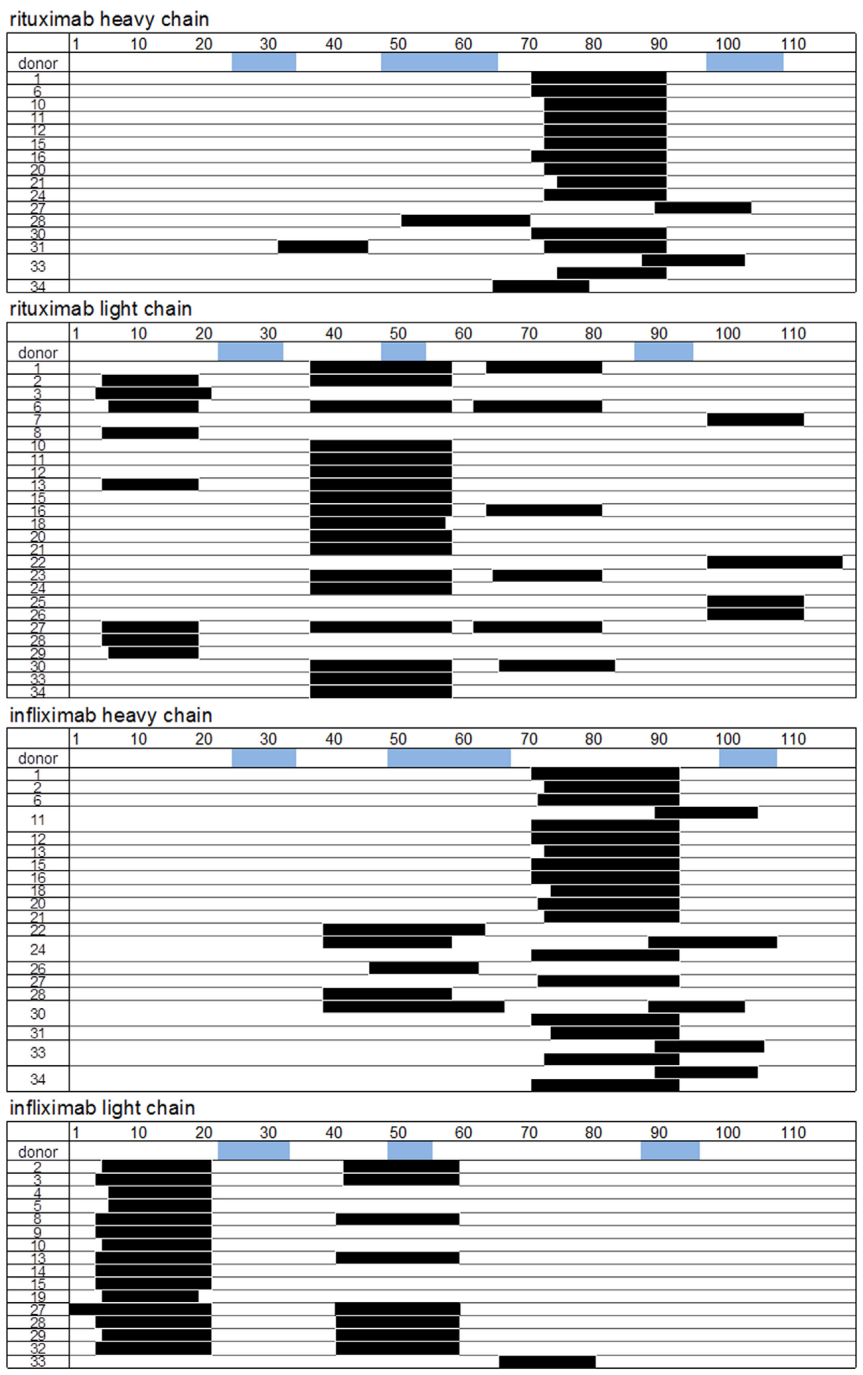
**Rituximab (Rtx) and infliximab (Ifx) peptides identified by MHC-associated peptide proteomics assay**. Human leukocyte antigen-DR molecules were immunopurified from dendritic cell loaded with Rtx or Ifx, and eluted peptides were identified by nano-high-performance liquid chromatography and electrospray ionization mass spectrometry (LC–ESI–MS/MS). Peptide sequences (black) are representative of the clusters found by each donor. Only donors with identified peptides were reported. Blue: CDRs.

Naturally presented HLA-DR-associated peptides were identified using the MAPPs assay (34–37) from monocyte-derived DCs of 34 healthy donors exposed to Rtx or Ifx. Peptides typically occur as multiple length variants, which share the same HLA-DR binding core and form a “cluster.” Five and four clusters were found in the VH and VL domains of Rtx, respectively (Figure 5). Each VH and VL domain of Ifx hosted four clusters. Four of the nine Rtx clusters and four of the eight Ifx clusters overlapped CDR regions, while the FR regions hosted about the half of the clusters. Many clusters of Ifx and Rtx encompassed the identified T cell epitopes.

### CD4 T Cell Epitopes Identified in the Healthy Donors Contribute to the T Cell Response to Ifx and Rtx in Patients Having Developed ADA

**Figure 6:**
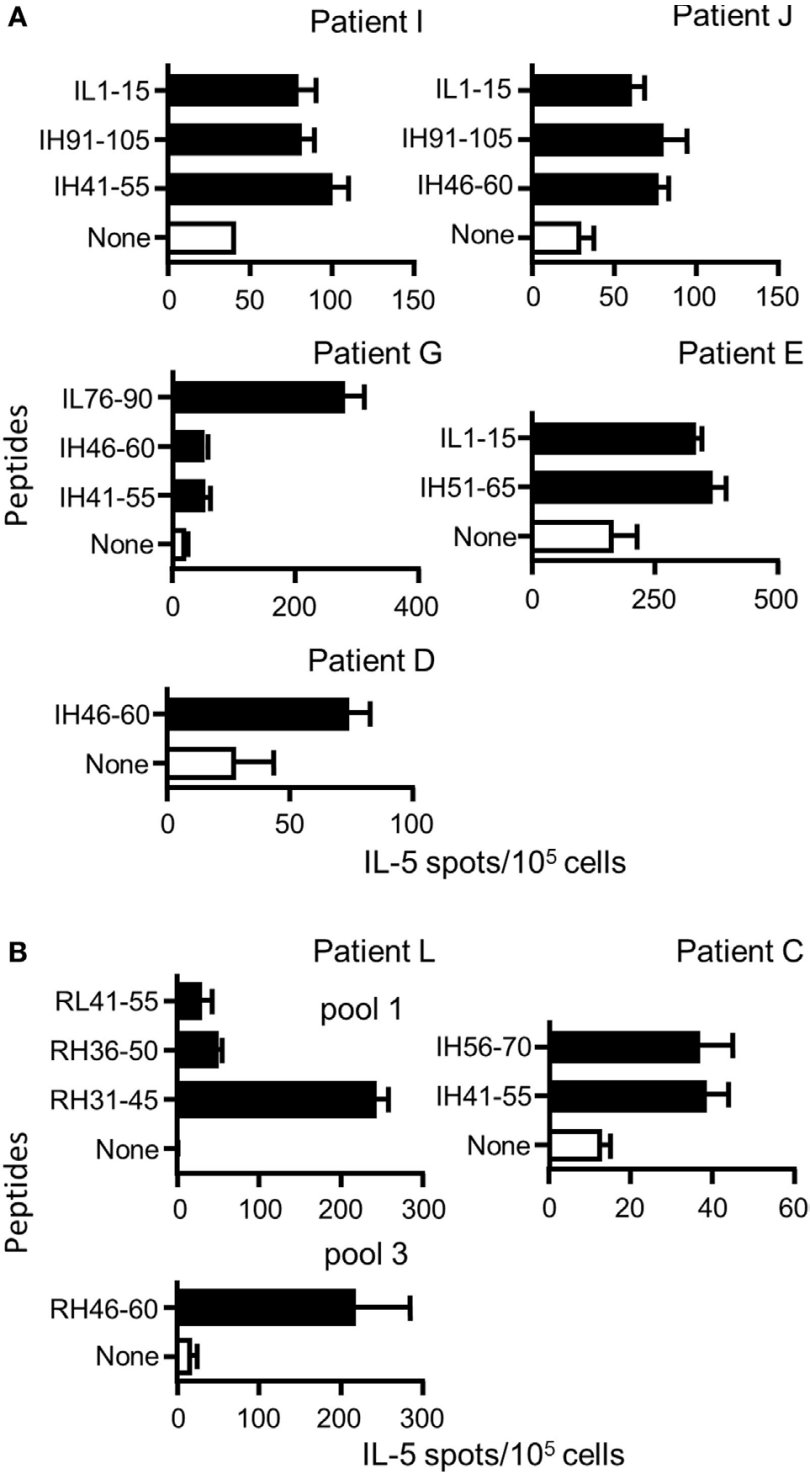
**Peptide specificity of T cells collected in patients with antidrug antibodies (ADAs) against rituximab (Rtx) or infliximab (Ifx)**. Peripheral blood mononuclear cells of patients with ADA against Rtx or Ifx were cultured with pools of Rtx and Ifx peptides for 10 days, and their peptide specificity was assessed by IL-5 ELISPOT using individual peptides **(A)** Ifx T cell epitopes already identified in the healthy donors composed the pool of peptides **(B)** All the peptides covering the entire sequences of the variable parts of Rtx or Ifx were introduced in the assay to evaluate the T cell response of the donors C and L. Only positive responses are presented [spot count that twofold higher in the presence of the peptide than in its absence, with a minimal difference of 25 spots and positivity in Student’s *t*-test (*P* < 0.05)].

We then investigated the CD4 T cell response in seven patients treated with Ifx (*n* = 6) or Rtx (*n* = 1) (one with granulomatous uveitis; five with Crohn’s disease, and one with rheumatoid arthritis) having developed ADAs against Ifx or Rtx (Table S5 in Supplementary Material). To detect memory CD4 T cells, PBMCs were submitted to a short-term T cell assay with only one step of *in vitro* stimulation instead of multiple rounds of antigenic stimulation as we did with healthy donors to detect naïve T cells. Accordingly, in this short-term T cell assay, PBMCs collected in three healthy donors did not generate a T cell response to the Rtx and Ifx T cell epitopes. PBMCs from five patients were cultured with a pool of the nine Ifx CD4 T cell epitopes for 10 days and then submitted to IL-5 ELISPOT using individual peptides (Figure 6A). All the patients responded to at least one peptide. Peptides IL1–15, IH41–55, and IH46–60 were common to three patients, while peptide IH91–105 was shared by two patients. Peptides IH51–65 and IL76–90 stimulated T cells in one patient, only. For two patients (L and C), sufficient amount of PBMCs was available to screen the peptide specificity of the T cells with the complete set of peptides and not with the T cell epitopes, only (Figure 6B). Four peptides (RL41–55, RH31–46, RH36–50, and RH46–60) among the 45 Rtx peptides gave rise to a T cell response for patient L. Two peptides (IH56–70 and IH41–55) from a total of the 46 Ifx peptides were active for patient C. All the peptides active in patients C and L are part of the Ifx and Rtx T cell epitopes we identified from the healthy donors.

**Figure 7:**
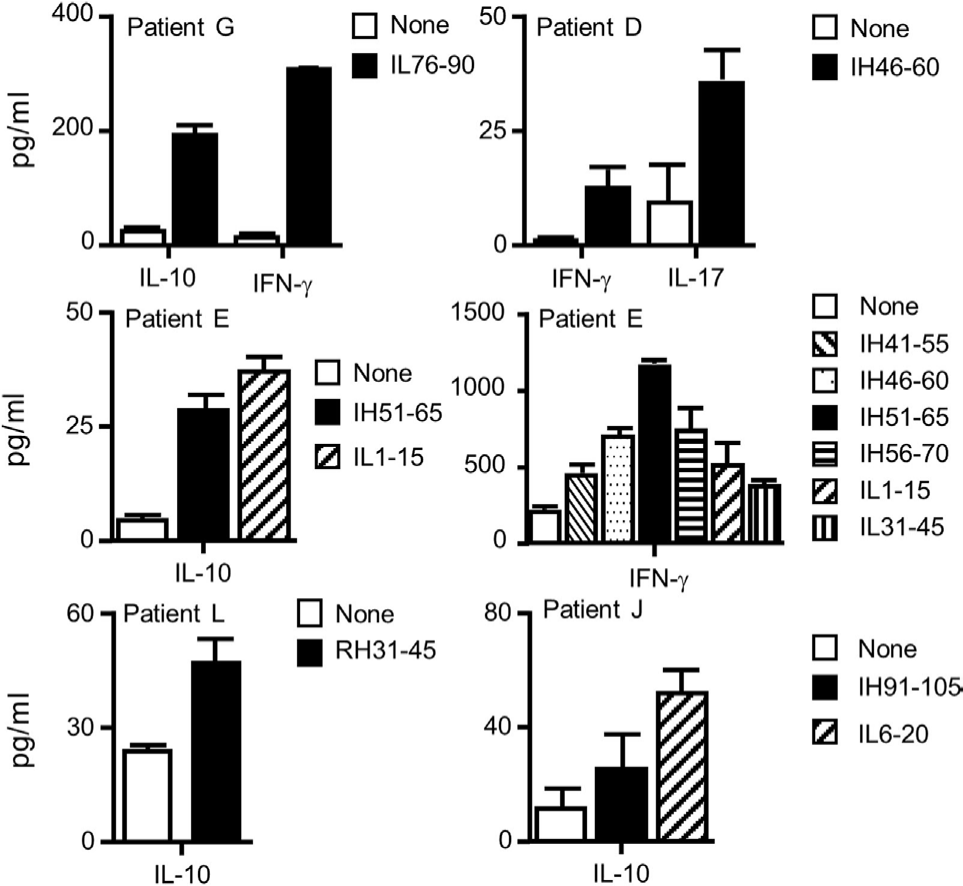
**Cytokine profiles of the T cells collected from patients with antidrug antibodies against rituximab or infliximab**. Supernatants of IL-5 ELISPOT plates were submitted to Luminex cytokine assays (R&D) for six different cytokines (IL-2, IL-4, IL-10, IL-13, IL-17, and interferon-γ). Only positive responses as defined in the Section “Materials and Methods” are presented.

Finally, supernatants of the ELISPOT plates were submitted to multiplex cytokine assay (Figure 7). Under the assay conditions, IL-2, IL-4, and IL-13 were not detected in any of the patients. Besides IL-5, PBMCs of patients L and J secreted IL-10, while patients E and G secreted IL-10 and IFN-γ. Patient D developed a T cell response characterized by the secretion of IFN-γ and IL-17, while a high level of IFN-γ secretion supported by multiple peptides was found for patient E. Altogether, we demonstrated that four Rtx and eight Ifx T cell epitopes identified in the healthy donors participated to the T cell responses elicited in patients having developed ADA against Rtx or Ifx. The T cell responses to Rtx and Ifx exhibited different patterns of secretion of cytokines, including IL-5, IFN-γ, IL-10, and IL-17.

## Discussion

**Figure 8:**
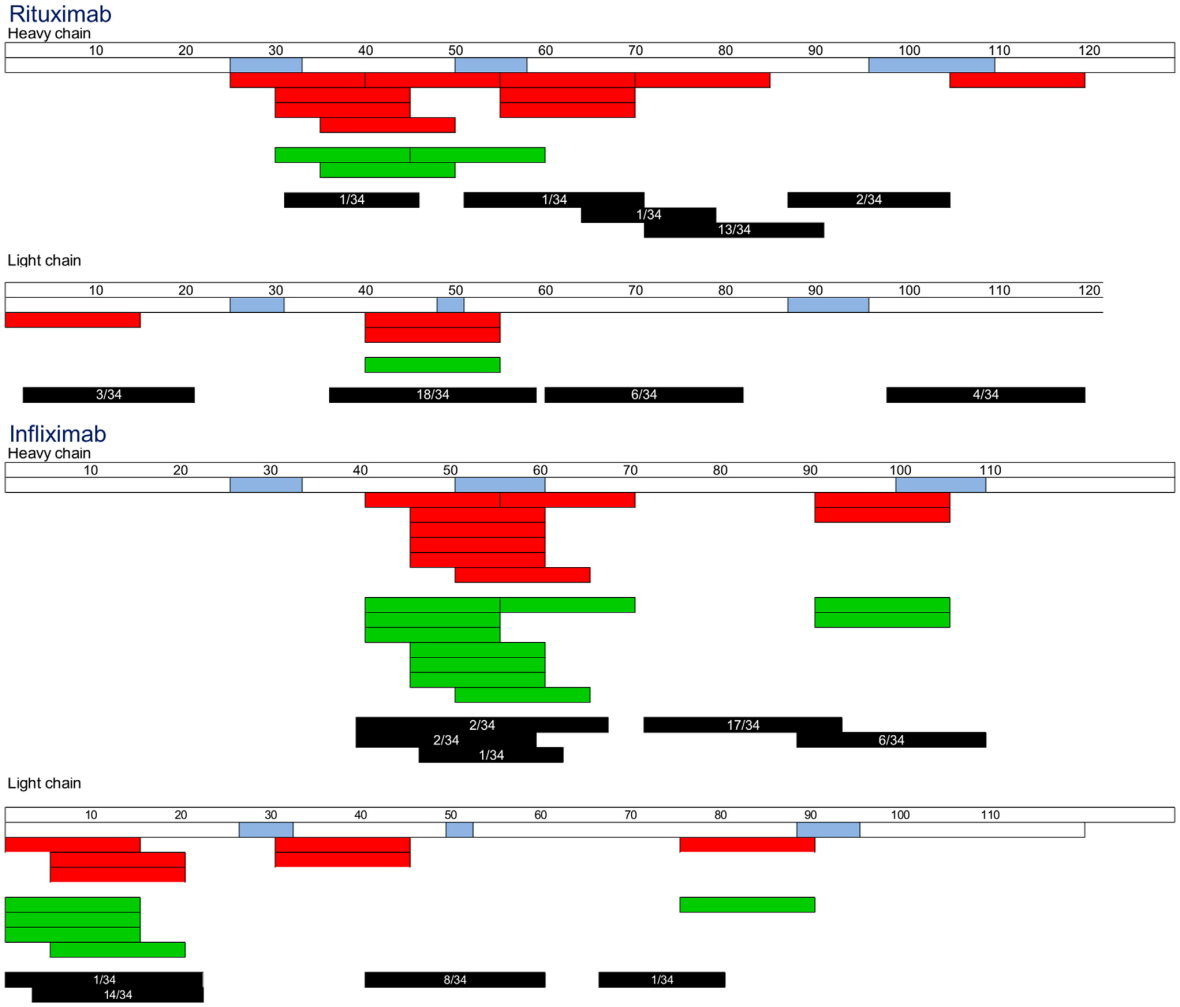
**Maps of the T cell epitopes of rituximab and infliximab**. T cell epitope sequences identified using cells collected in healthy donors (red) or in patients with antidrug antibodies (green) were reported, each bar corresponding to an individual response. Black: cluster identified by MHC-associated peptide proteomics assay. Occurrence of each cluster among the donors tested is indicated inside each bar.

With the perspective to anticipate immunogenicity of therapeutic antibodies, we identified CD4 T cell epitopes of the immunogenic chimeric antibodies Ifx (8–10) and Rtx (5–7) from healthy donors. We evaluated the capacity of the CD4 T cell epitopes identified in the healthy donors to stimulate T cells collected from patients with ADA. We evaluated the binding affinity of overlapping VH and VL peptides to preponderant HLA-DR molecules and identified naturally presented HLA-DR-associated peptides displayed by human DCs. The main results of this comprehensive analysis of the CD4 T cell epitopes of Rtx and Ifx are summarized in Figure 8.

Although the immunogenicity of therapeutic antibodies such as Ifx and Rtx is a limitation for their clinical use, their CD4 T cell epitopes have not yet been identified. We therefore isolated specific T cell lines from CD4 T lymphocytes collected from healthy donors by multiple rounds of *in vitro* antigenic stimulation (28), while memory CD4 T cell response was investigated in ADA patients using a short-term T cell assay. For each antibody, the number of T cell lines derived from each healthy donor ranged from 0 to 6, in agreement with the very low frequency of Ifx- and Rtx-specific T cells preexisting in the blood of healthy donors (28). Nine T cell epitopes were found in both antibodies, spreading over 25–58% of the VH and VL sequences (Figure 8). They mainly overlap CDR regions, but RH71–85, RL1–15, IL1–15, and IL6–20 are entirely embedded in the FR regions. The wide spread of the T cell epitopes along the variable parts of Rtx and Ifx and not only the CDR is consistent with their murine origin as their FR regions could be recognized as foreign by the human immune system (3). The location of Ifx and Rtx T cell epitopes also seems to rely on HLA binding affinity, as most of the T cell epitopes appeared to be associated with strong or moderate peptide binding to HLA molecules (Table 1). However, as previously observed for other antigens (33, 38), including therapeutic proteins (25), not all of HLA binders stimulated T cells (Figure 4). Naturally presented peptide sequences retrieved from the MAPPs assay (34–37) span a more restricted portion of the VH and VL sequences as compared to HLA-DR binding regions. In many cases, sequences of peptides eluted from HLA-DR molecules from human DCs nicely match with the T cell epitopes (Figure 8). Only three T cell epitopes (RH106–120, IL 31–45, and IL76–90) are not included in the peptides identified by MAPPs. The assays were performed on different donor sets, which might be a reason for the mismatches. Of particular interest are immunoprevalent T cell epitopes (39) that are common to multiple donors. Immunoprevalence of Ifx IH46–60 and Rtx RL41–55 seemed to result from their capacity to bind multiple HLA class II molecules corresponding to promiscuous T cell epitopes. Rtx RL41–55 was also naturally presented by more than half of the donors tested *via* MAPPs. In contrast, the immunoprevalent Rtx peptide RH56–70 bound strongly only to the HLA-DRB1*11:01 molecule, and in the MAPPs assay the corresponding sequence region was displayed by only one donor who carried the same allele. Accordingly, the three donors responding to this peptide were genotyped HLA-DRB1*11:01. Together, our data from HLA-DR binding experiments, natural peptide presentation experiments, and T cell epitope mapping experiments show that the presented peptide repertoire is smaller than the peptide binding repertoire, and that the T cell epitope repertoire appears to be smaller than the presented peptide repertoire.

We also provide new insights into the specificity of the T cell response to Rtx and Ifx in patients having developed ADAs. All patients generated a T cell response to either Rtx or Ifx peptides, confirming that the ADA response is a T-cell-dependent process (16–20). Eight out of nine Ifx T cell epitopes and four out of nine Rtx T cell epitopes, which we identified in healthy blood donors contributed to the T cell response in patients having developed ADAs. As the healthy donors have never been treated with these antibodies, the T cell epitopes we identified from the cells collected in these donors should emerge from naïve T cell cells (28). Our data are therefore consistent with previous observations in animal models using HLA tetramers (40) and in vaccinated donors (41, 42) showing that memory CD4 T cell response is shaped by the naïve repertoire. Accordingly, immunogenic therapeutic antibodies including Ifx and Rtx exhibit a larger repertoire of preexisting naïve CD4 T lymphocytes in healthy donors than most of the antibodies considered as non-immunogenic (28). T cell epitopes in therapeutic antibodies could be at least partly predicted by using cells collected from healthy donors (25–29). Such T cell assays allow therapeutic antibodies to be ranked for immunogenicity risk on the basis of their T cell epitope content. T cell epitopes could be removed from the antibody sequence to minimize their immunogenicity, as already proposed (19, 21).

Our findings also contribute to describe the diversity of the T cell response developed in patients with ADAs. Besides IL-5, we also detected secretion of IFN-γ, IL-10, and IL-17, but with different patterns of secretion across the patients. These data illustrate the heterogeneity of the T cell response to chimeric antibodies across inflammatory cytokines and the immunosuppressive cytokine IL-10 (43). Using the identified T cell epitopes, further studies would help to understand the diversity of immune responses, including the allergic response (2), to classify patients with and without an ADA response and to identify early markers of ADA response.

In conclusion, we identified the CD4 T cell epitopes of Ifx and Rtx from healthy donors and demonstrated their relevance to participate to the T cell response developed in patients with ADAs. We also compared them to HLA binding data as well as to the naturally presented peptide repertoire. These data contribute to the understanding of the root cause of immunogenicity of chimeric antibodies. They could help to improve the diagnostic of ADA responses (2) and the selection of therapeutic antibodies with low immunogenicity by removing the T cell epitopes from the initial sequence.

## Ethics Statement

This study was carried out in accordance with the recommendations of the guidelines of the etablissement farnçais du sang (EFS), Comité d’ethique et deontologie de EFS with written informed consent from all subjects. All subjects gave written informed consent in accordance with the Declaration of Helsinki. The protocol was approved by le Comité d’ethique et deontologie de EFS.

## Author Contributions

MH, SM, AK, SS, XM, and BM designed the study and interpreted the data; BM, AK, MH, and SM wrote the manuscript; MH, SM, AGd, AGo, and NS performed the experiments; BM, MP, and AK supervised the experiments; and XM, FC, and CM-R selected the patients and supervised the collection of blood samples. All authors revised the work and gave final approval of the version to be published.

## Conflict of Interest Statement

AK and SS are full-time employees of Novartis. The other authors declare no conflict of interest. The reviewer, ER, and handling Editor declared their shared affiliation, and the handling Editor states that the process nevertheless met the standards of a fair and objective review.

## Abbreviations

DCs, dendritic cells; F, female; HLA, human leukocyte antigen; HSA, human serum albumin; IFN-γ, interferon-γ; KLH, keyhole limpet hemocyanin; Ifx, infliximab; MAPPs, MHC-associated peptide proteomics; MS, mass spectrometry; PBMCs, peripheral blood mononuclear cells; Rtx, rituximab.
