# Supplementary material for: Characterization of CD4 T Cell Epitopes of Infliximab and Rituximab Identified from Healthy Donors
Source: Front Immunol. 2017 May 5;8:500. doi: 10.3389/fimmu.2017.00500 (PMC5418239; doi:10.3389/fimmu.2017.00500)
Supplement: Supplementary file 1 [file data_sheet_1.docx]

# Supplementary materials

| Ifx T cell experiments | | | Rtx T cell experiments | | |
| --- | --- | --- | --- | --- | --- |
| Donors | HLA-DRB1 Typing | | Donors | HLA-DRB1 Typing | |
| 160 | 04:01 | 07:01 | 66 | 09:01 | 13:01 |
| 209 | 04:01 | 04:01 | 90 | 07:01 | 11:01 |
| 221 | 01:01 | 07:01 | 91 | 07:01 | 16:01 |
| 222 | 01:01 | 01:02 | 97 | 04:01 | 07:01 |
| 226 | 15:01 | 15:01 | 113 | 03:01 | 11:01 |
| 241 | 04:02 | 15:01 | 135 | 03:01 | 15:01 |
| 244 | 01:01 | 13:02 | 136 | 11:01 | 14:01 |
| 247 | 11:01 | 16:01 | 142 | 11:01 | 15:02 |
| 248 | 07:01 | 11:01 | 201 | 16:01 | 13:01 |
| 225 | 01:02 | 13:01 | 212 | 15:01 | 15:01 |
| 250 | 03:01 | 07:01 | 241 | 04:02 | 15:01 |
| 251 | 07:01 | 09:01 | 243 | 03:01 | 08:01 |
| 261 | 03:01 | 11:04 | 244 | 01:01 | 13:02 |
| 260 | 11:01 | 13:02 | 262 | 01:01 | 08:01 |
| 279 | 03:02 | 04:02 | 265 | 01:01 | 01:01 |

Table S1: HLA-DRB1 typing of healthy donors used to derive T cell lines raised against Ifx and Rtx

HLA-DR genotyping was performed using the AllSet+™ Gold SSP DRB1 High-Resolution Kit (Invitrogen, Carlsbad, CA) after DNA extraction from PBMCs with the NucleoSpin Blood L Kit (Macherey Nagel).

| Peptide | Sequence | Pool |
| --- | --- | --- |
| IH1-15  IH6-20  IH11-25  IH16-30  IH21-35  IH26-40  IH31-45  IH36-50 | EVKLEESGGGLVQPG  ESGGGLVQPGGSMKL  LVQPGGSMKLSCVAS  GSMKLSCVASGFIFS  SCVASGFIFSNHWMN  GFIFSNHWMNWVRQS  NHWMNWVRQSPEKGL  WVRQSPEKGLEWVAE | Pool 1 |
| IH41-55  IH46-60  IH51-65  IH56-70  IH61-75  IH66-80  IH71-85  IH76-90 | PEKGLEWVAEIRSKS  EWVAEIRSKSINSAT  IRSKSINSATHYAES  INSATHYAESVKGRF  HYAESVKGRFTISRD  VKGRFTISRDDSKSA  TISRDDSKSAVYLQM  DSKSAVYLQMTDLRT | Pool 2 |
| IH81-95  IH86-100  IH91-105  IH96-110  IH101-115  IH106-120  IH111-125  IH116-130 | VYLQMTDLRTEDTGV  TDLRTEDTGVYYCSR  EDTGVYYCSRNYYGS  YYCSRNYYGSTYDYW  NYYGSTYDYWGQGTT  TYDYWGQGTTLTVSS  GQGTTLTVSSASTKG  LTVSSASTKGPSVFP | Pool 3 |
| IL1-15  IL6-20  IL11-25  IL16-30  IL21-35  IL26-40  IL31-45  IL36-50 | DILLTQSPAILSVSP  QSPAILSVSPGERVS  LSVSPGERVSFSCRA  GERVSFSCRASQFVG  FSCRASQFVGSSIHW  SQFVGSSIHWYQQRT  SSIHWYQQRTNGSPR  YQQRTNGSPRLLIKY | Pool 4 |
| IL41-55  IL46-60  IL51-65  IL56-70  IL61-75  IL66-80  IL71-85 | NGSPRLLIKYASESM  LLIKYASESMSGIPS  ASESMSGIPSRFSGS  SGIPSRFSGSGSGTD  RFSGSGSGTDFTLSI  GSGTDFTLSINTVES  FTLSINTVESEDIAD | Pool 5 |
| IL76-90  IL81-95  IL86-100  IL91-105  IL96-110  IL101-115  IL106-120 | NTVESEDIADYYCQQ  EDIADYYCQQSHSWP  YYCQQSHSWPFTFGS  SHSWPFTFGSGTNLE  FTFGSGTNLEVKRTV  GTNLEVKRTVAAPSV  VKRTVAAPSVFIFPP | Pool 6 |

Table S3: sequence of infliximab peptides

| Peptide | Sequence | Pool |
| --- | --- | --- |
| RH1-15  RH6-20  RH11-25  RH16-30  RH21-35  RH26-40  RH31-45  RH36-50 | QVQLQQPGAELVKPG  QPGAELVKPGASVKM  LVKPGASVKMSCKAS  ASVKMSCKASGYTFT  SCKASGYTFTSYNMH  GYTFTSYNMHWVKQT  SYNMHWVKQTPGRGL  WVKQTPGRGLEWIGA | Pool 1 |
| RH41-55  RH46-60  RH51-65  RH56-70  RH61-75  RH66-80  RH71-85  RH76-90 | PGRGLEWIGAIYPGN  EWIGAIYPGNGDTSY  IYPGNGDTSYNQKFK  GDTSYNQKFKGKATL  NQKFKGKATLTADKS  GKATLTADKSSSTAY  TADKSSSTAYMQLSS  SSTAYMQLSSLTSED | Pool 2 |
| RH81-95  RH86-100  RH91-105  RH96-110  RH101-115  RH106-120  RH111-125  RH116-130 | MQLSSLTSEDSAVYY  LTSEDSAVYYCARST  SAVYYCARSTYYGGD  CARSTYYGGDWYFNV  YYGGDWYFNVWGAGT  WYFNVWGAGTTVTVS  WGAGTTVTVSAASTK  TVTVSAASTKGPSVF | Pool 3 |
| RL1-15  RL6-20  RL11-25  RL16-30  RL21-35  RL26-40  RL31-45 | IVLSQSPAILSASP  QSPAILSASPGEKVT  LSASPGEKVTMTCRA  GEKVTMTCRASSSVS  MTCRASSSVSYIHWF  SSSVSYIHWFQQKPG  YIHWFQQKPGSSPKP | Pool 4 |
| RL36-50  RL41-55  RL46-60  RL51-65  RL56-70  RL61-75  RL66-80 | QQKPGSSPKPWIYAT  SSPKPWIYATSNLAS  WIYATSNLASGVPVR  SNLASGVPVRFSGSG  GVPVRFSGSGSGTSY  FSGSGSGTSYSLTIS  SGTSYSLTISRVEAE | Pool 5 |
| RL71-85  RL76-90  RL81-95  RL86-100  RL91-105  RL96-110  RL101-115 | SLTISRVEAEDAATY  RVEAEDAATYYCQQW  DAATYYCQQWTSNPP  YCQQWTSNPPTFGGG  TSNPPTFGGGTKLEI  TFGGGTKLEIKRTVA  TKLEIKRTVAAPSVF | Pool 6 |

Table S4: sequence of rituximab peptides

| Patient | Sex | Age (yr) | Disease | Treatment | Drug (µg/ml) | ADA level (U/ml) |
| --- | --- | --- | --- | --- | --- | --- |
| C | M | 12 | Uveitis. | Ifx | <0.1 | 63 |
| D | F | 57 | Crohn’s. | Ifx | 0 | 61 |
| E | F | 71 | Crohn’s | Ifx | <0.1 | >200 |
| G | M | 19 | Crohn’s | Ifx | 0.13 | >200 |
| I | F | 23 | Crohn’s | Ifx | 0.11 | >200 |
| J | M | 59 | Crohn’s. | Ifx | <0.1 | 95 |
| L | F | 56 | RA. | Rtx | <2 | >100 |

Table S5: Characteristics of patients having developed ADA against Ifx or Rtx.

Patients were under treatment with either Ifx or Rtx and have developed ADA. Levels of circulating Ifx or Rtx and of anti-drug antibodies (ADAs) specific for each antibody were quantified using bridging ELISA kits (Theradiag, Croissy Beaubourg, France). RA., rheumatoid arthritis

**Legend supplemental figure**

Figure S2: Examples of individual T cell response to KLH

CD4 T cell lines were generated *in vitro* by four weekly rounds of stimulation with autologous DCs loaded with KLH. Their specificity was investigated by IFN-γ ELISPOT using autologous DCs alone (white) or DCs previously loaded with KLH (black). Each panel reports the KLH-specific T cell lines of one donor.
