# Supplementary figures and images for: Characterization of CD4 T Cell Epitopes of Infliximab and Rituximab Identified from Healthy Donors
Source: Front Immunol. 2017 May 5;8:500. doi: 10.3389/fimmu.2017.00500 (PMC5418239; doi:10.3389/fimmu.2017.00500)

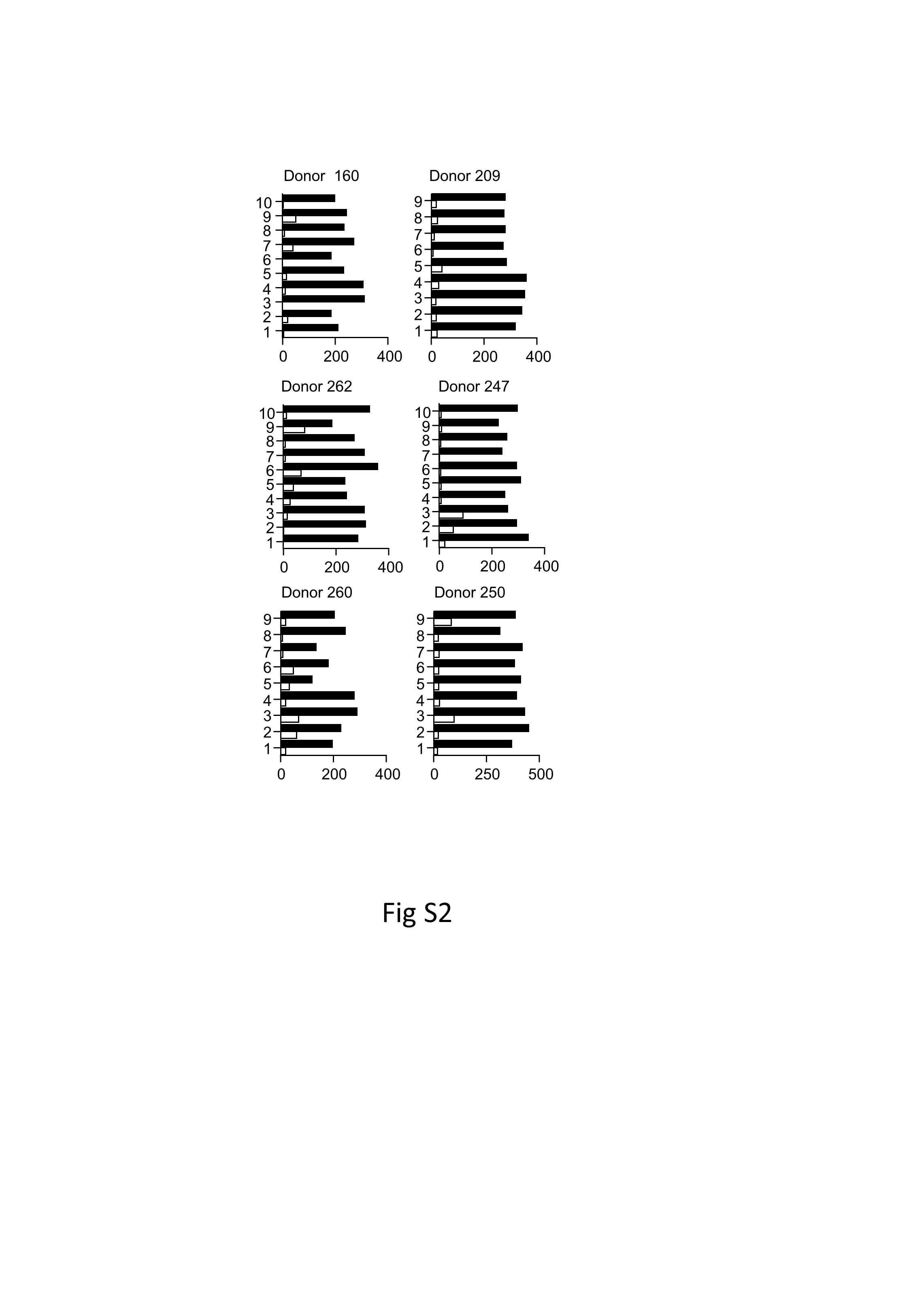

Supplement: Supplementary file 2 [file image_1.tif]
